# Supplementary figures and images for: MAGICAL: A multi-class classifier to predict synthetic lethal and viable interactions using protein-protein interaction network
Source: PLoS Comput Biol. 2024 Aug 26;20(8):e1012336. doi: 10.1371/journal.pcbi.1012336 (PMC12529998; doi:10.1371/journal.pcbi.1012336)

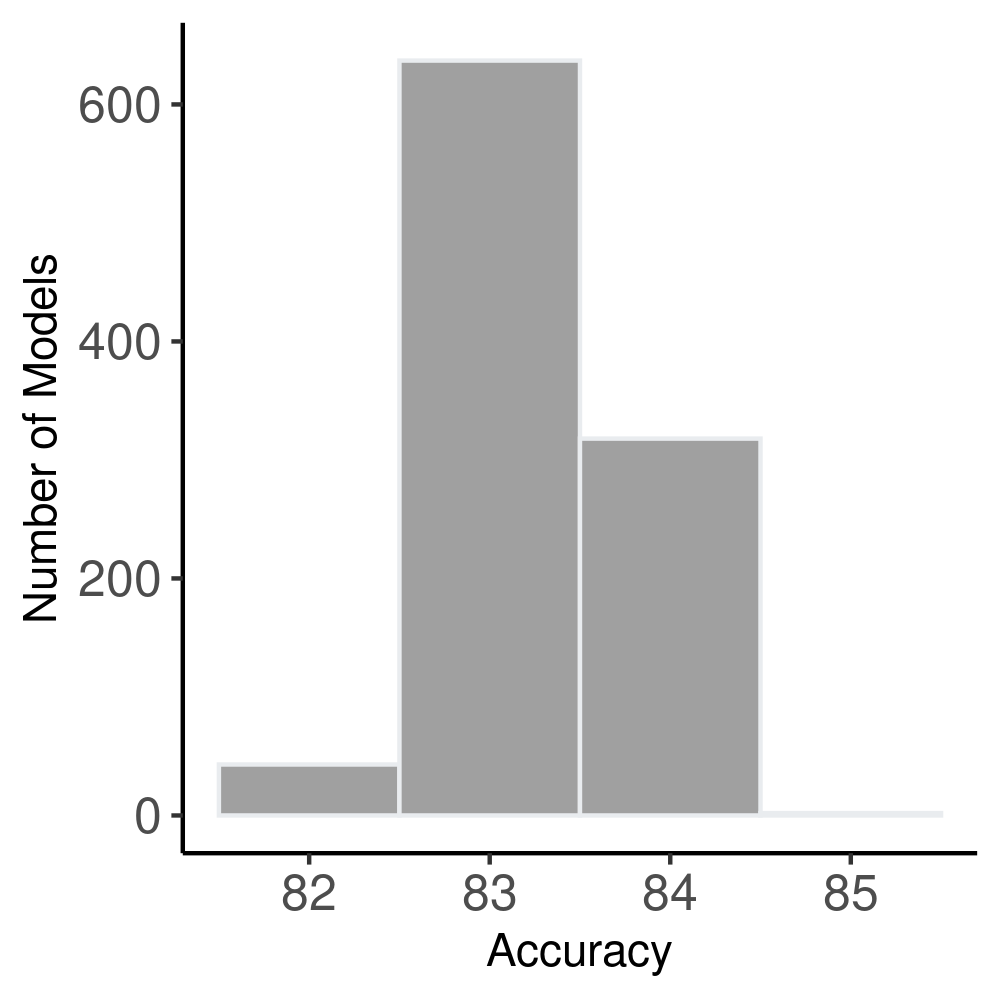

Supplement: S1 Fig — (TIFF) [file pcbi.1012336.s001.tiff]

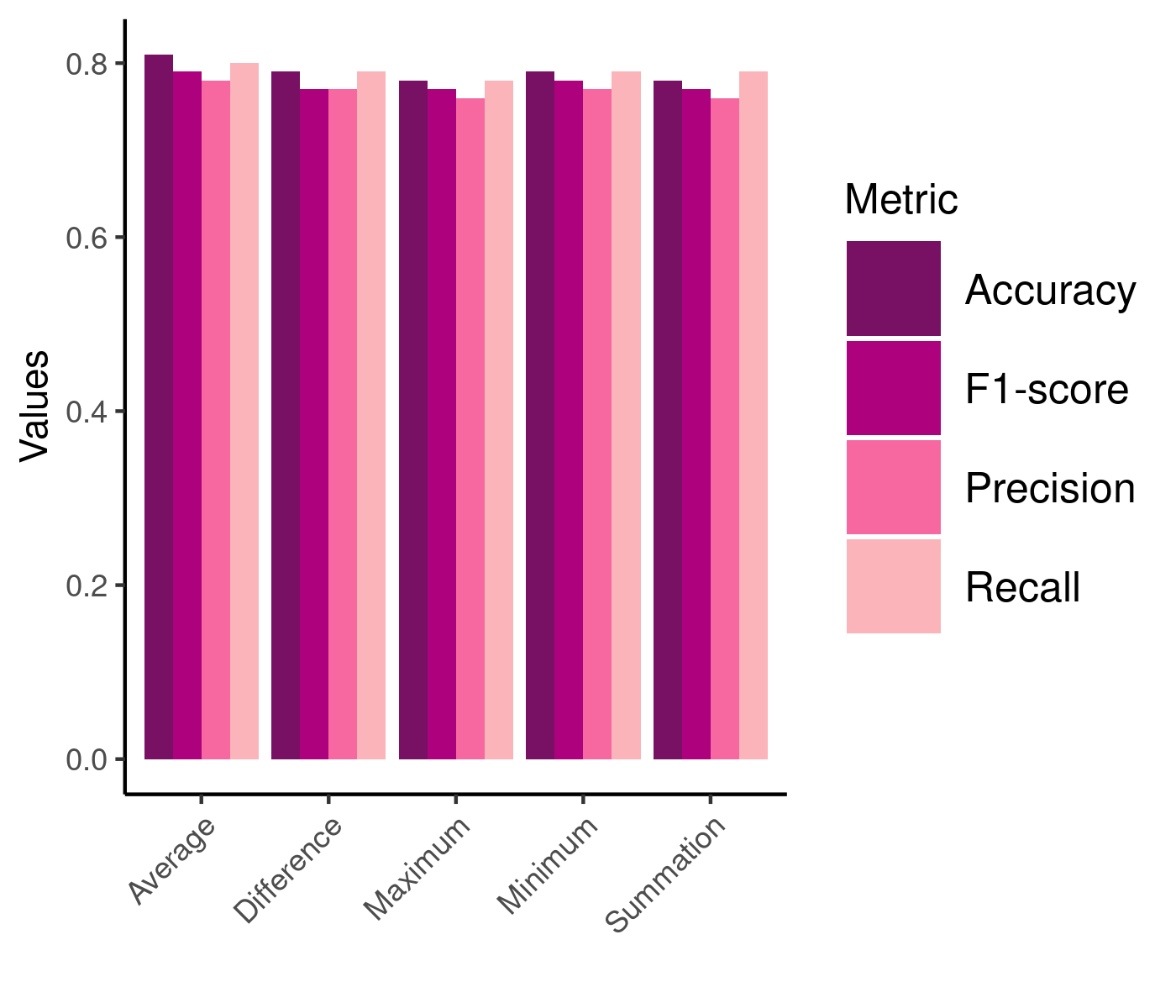

Supplement: S2 Fig — (TIFF) [file pcbi.1012336.s002.tiff]

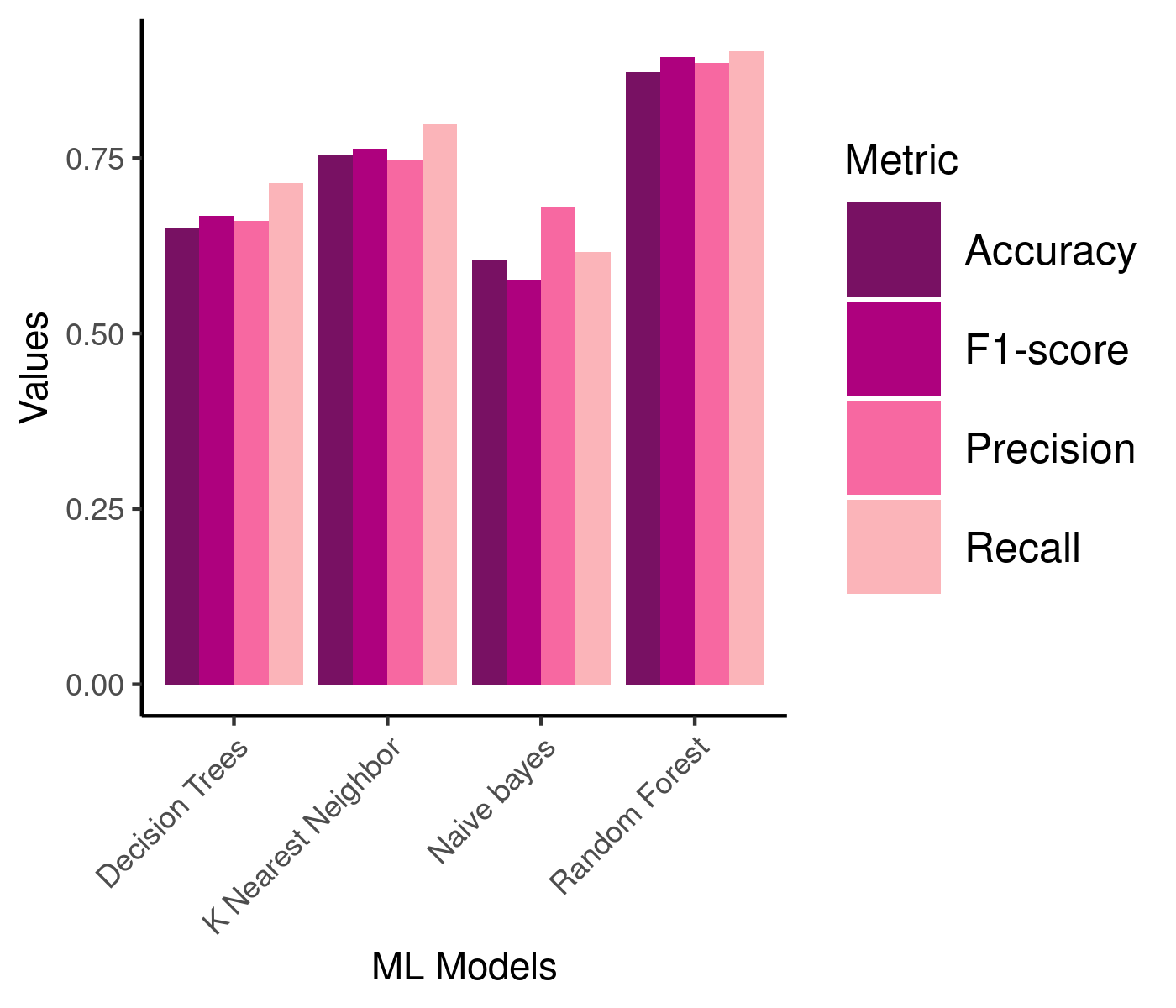

Supplement: S3 Fig — (TIFF) [file pcbi.1012336.s003.tiff]

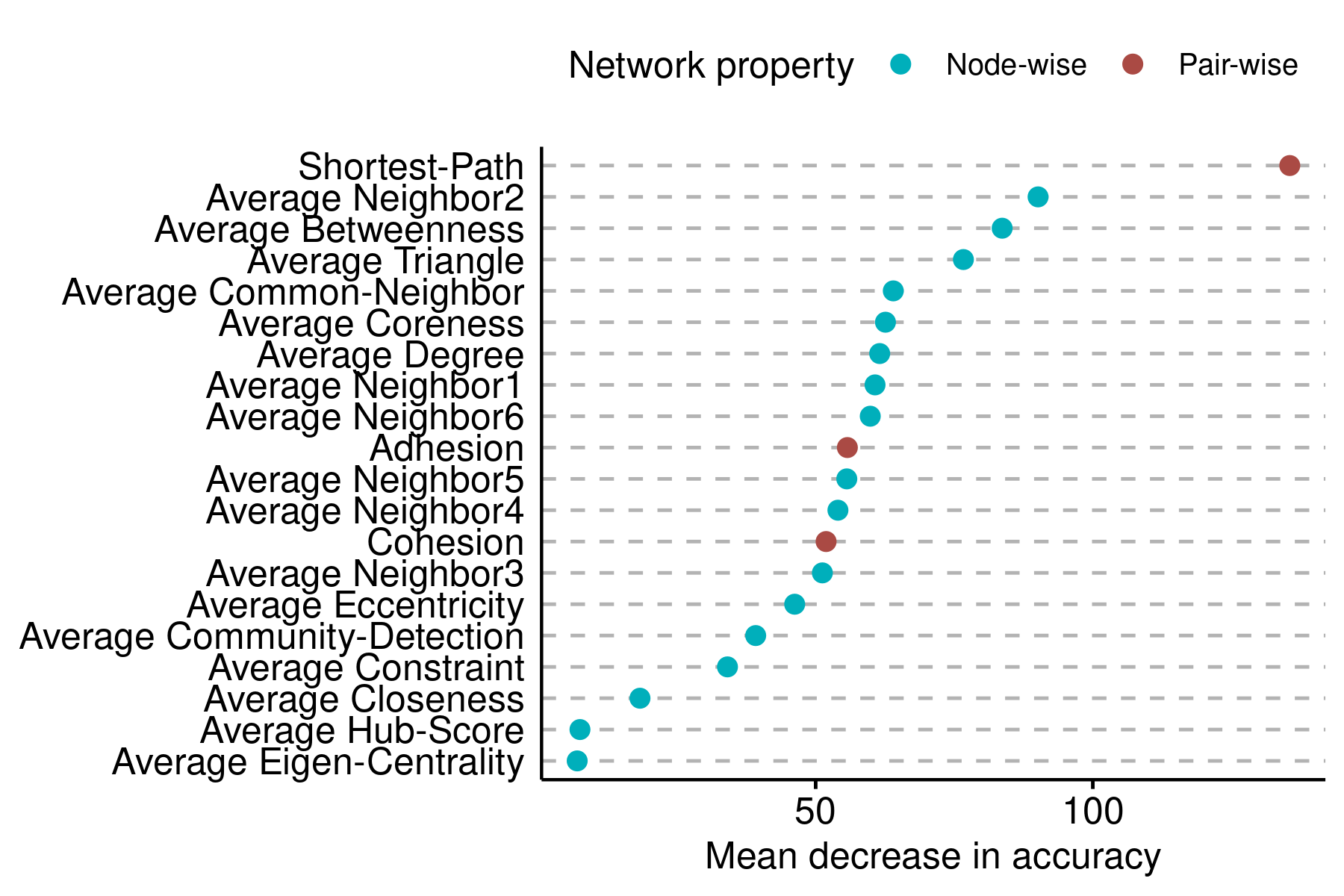

Supplement: S4 Fig — Note: The importance of the feature decreases from top to bottom. (TIFF) [file pcbi.1012336.s004.tiff]

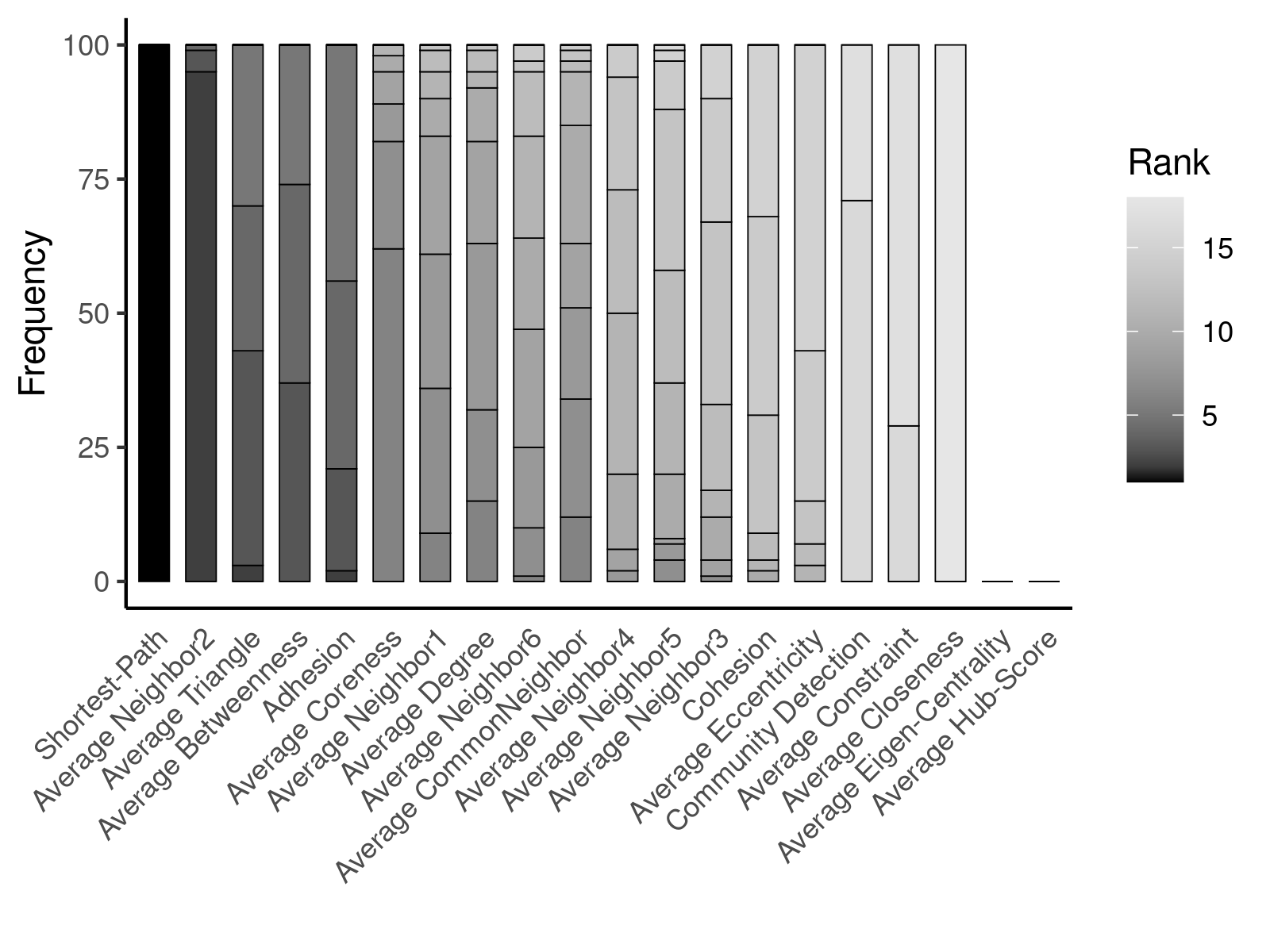

Supplement: S5 Fig — (TIFF) [file pcbi.1012336.s005.tiff]

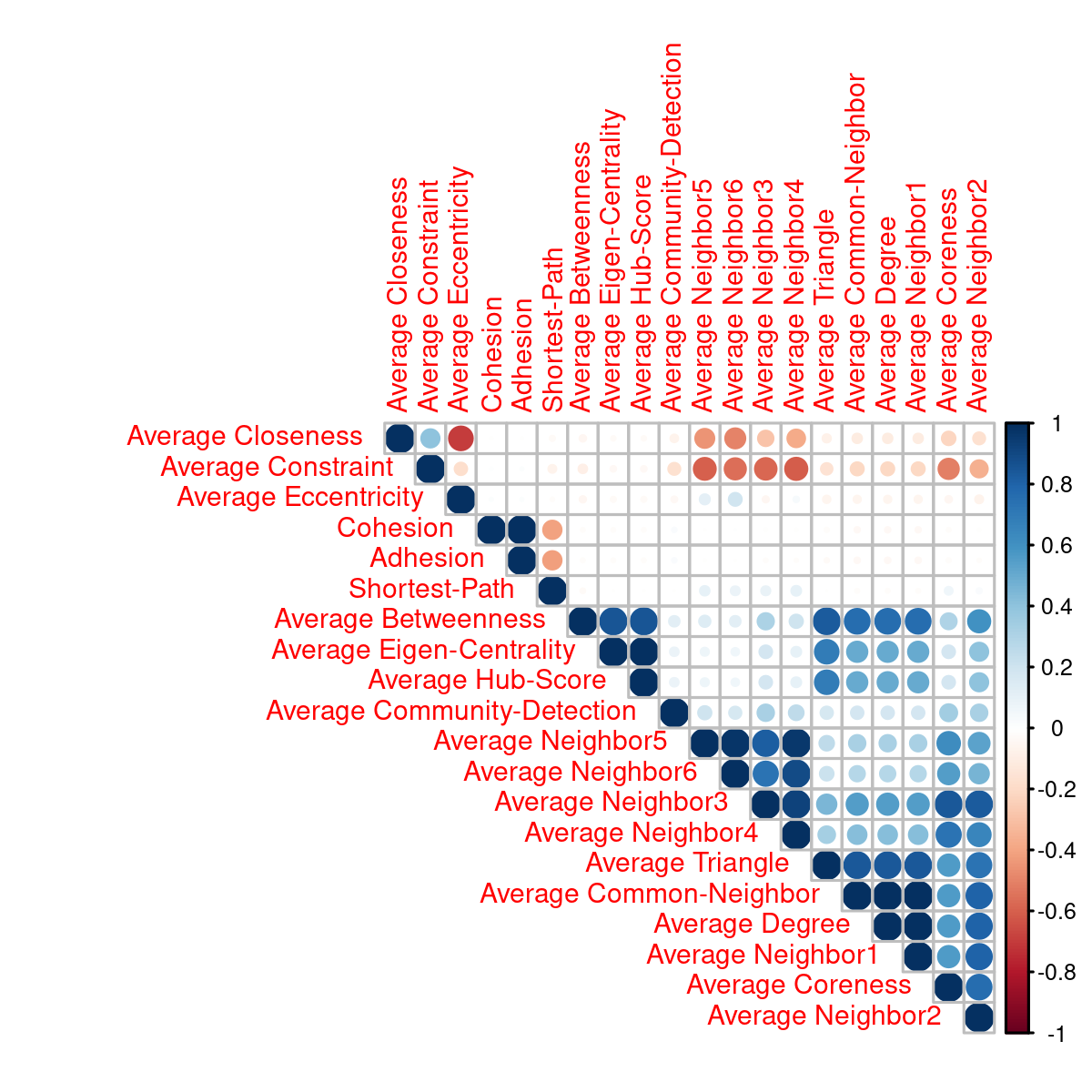

Supplement: S6 Fig — (Blue: positive correlation, Red: negative correlation). The size of the circles indicates the significance of the p-value for spearman correlation test. (TIFF) [file pcbi.1012336.s006.tiff]

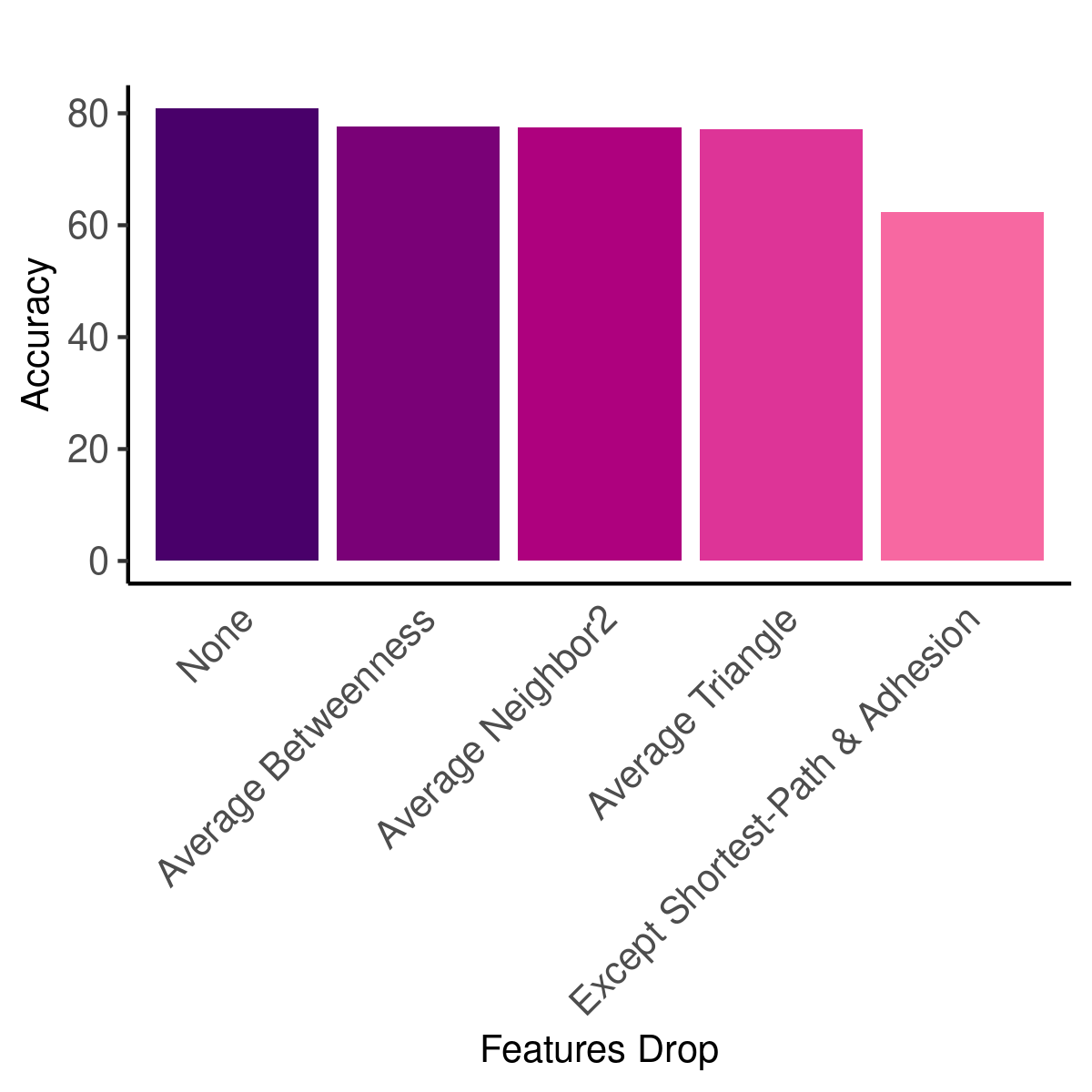

Supplement: S7 Fig — (TIFF) [file pcbi.1012336.s007.tiff]

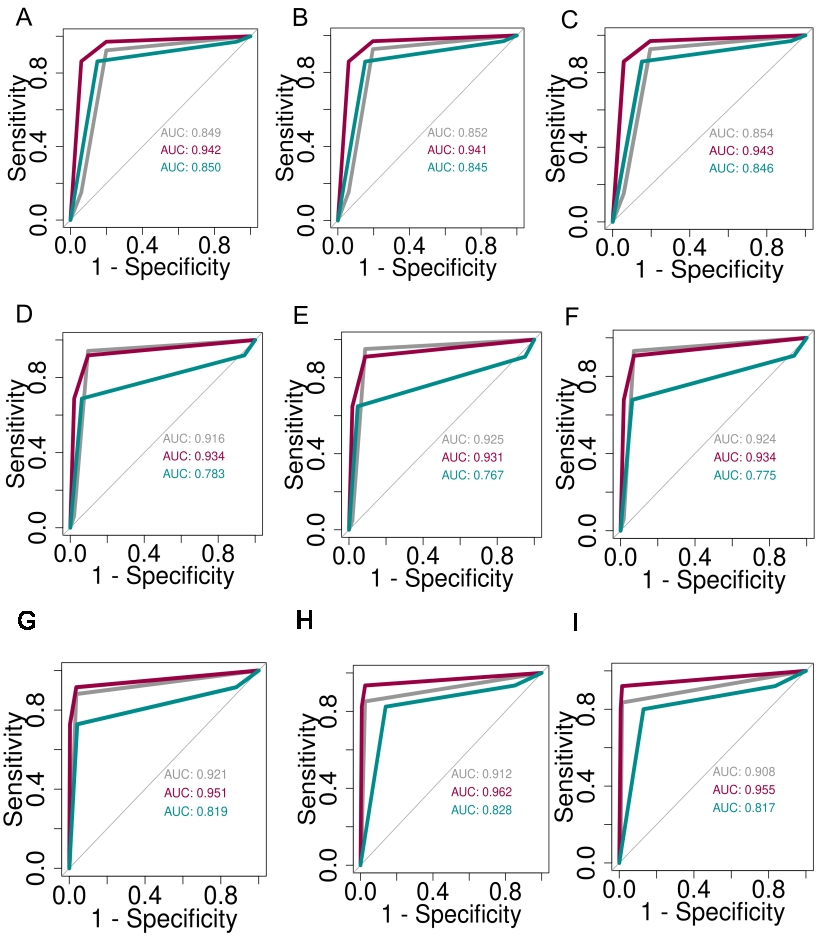

Supplement: S8 Fig — For each plot, we notice a lower value of False Positive Rate and a higher value of True Positive Rate. (TIFF) [file pcbi.1012336.s008.tiff]

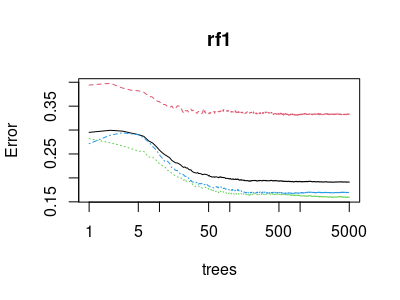

Supplement: S9 Fig — (TIFF) [file pcbi.1012336.s009.tiff]

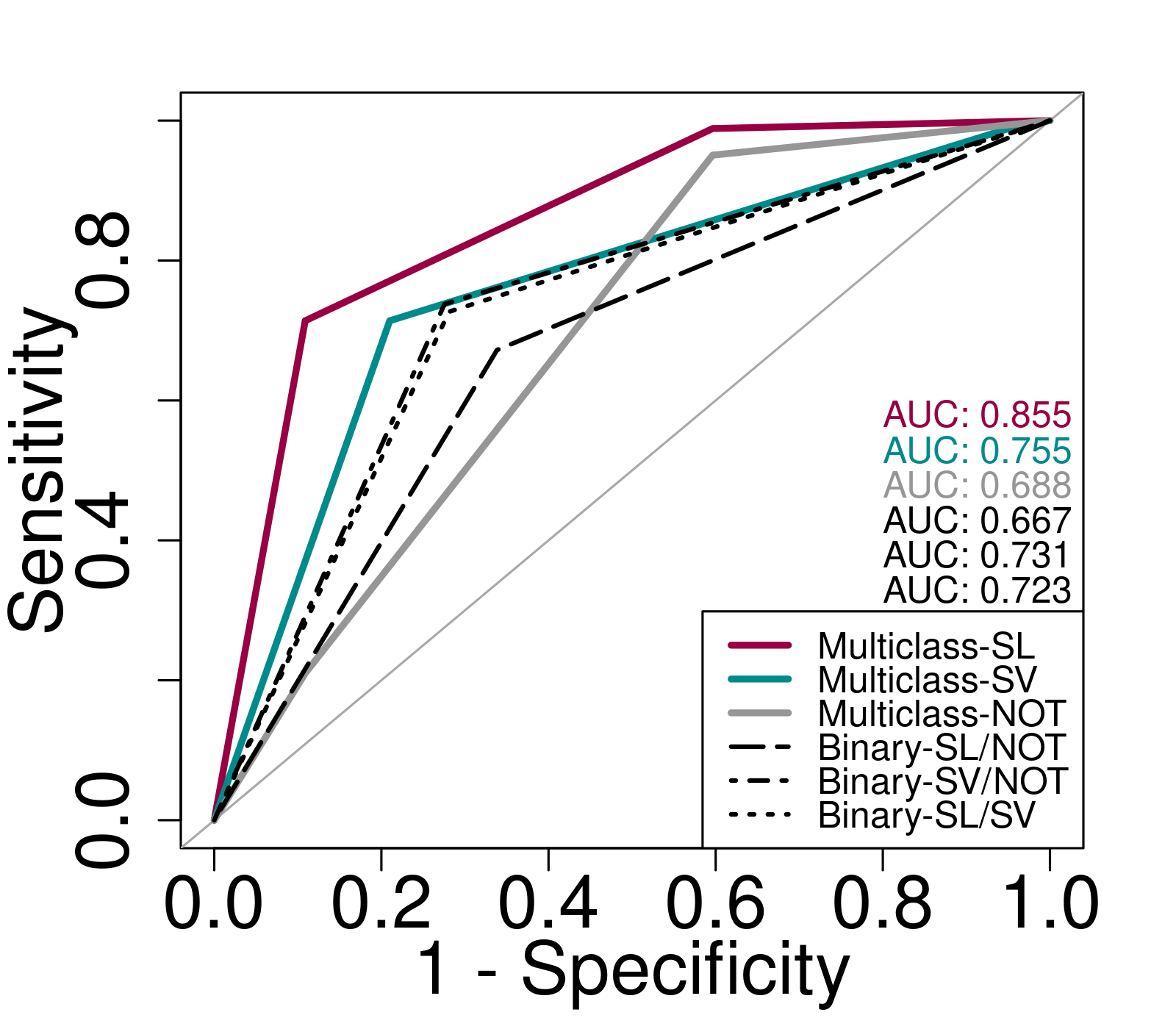

Supplement: S10 Fig — (TIFF) [file pcbi.1012336.s010.tiff]

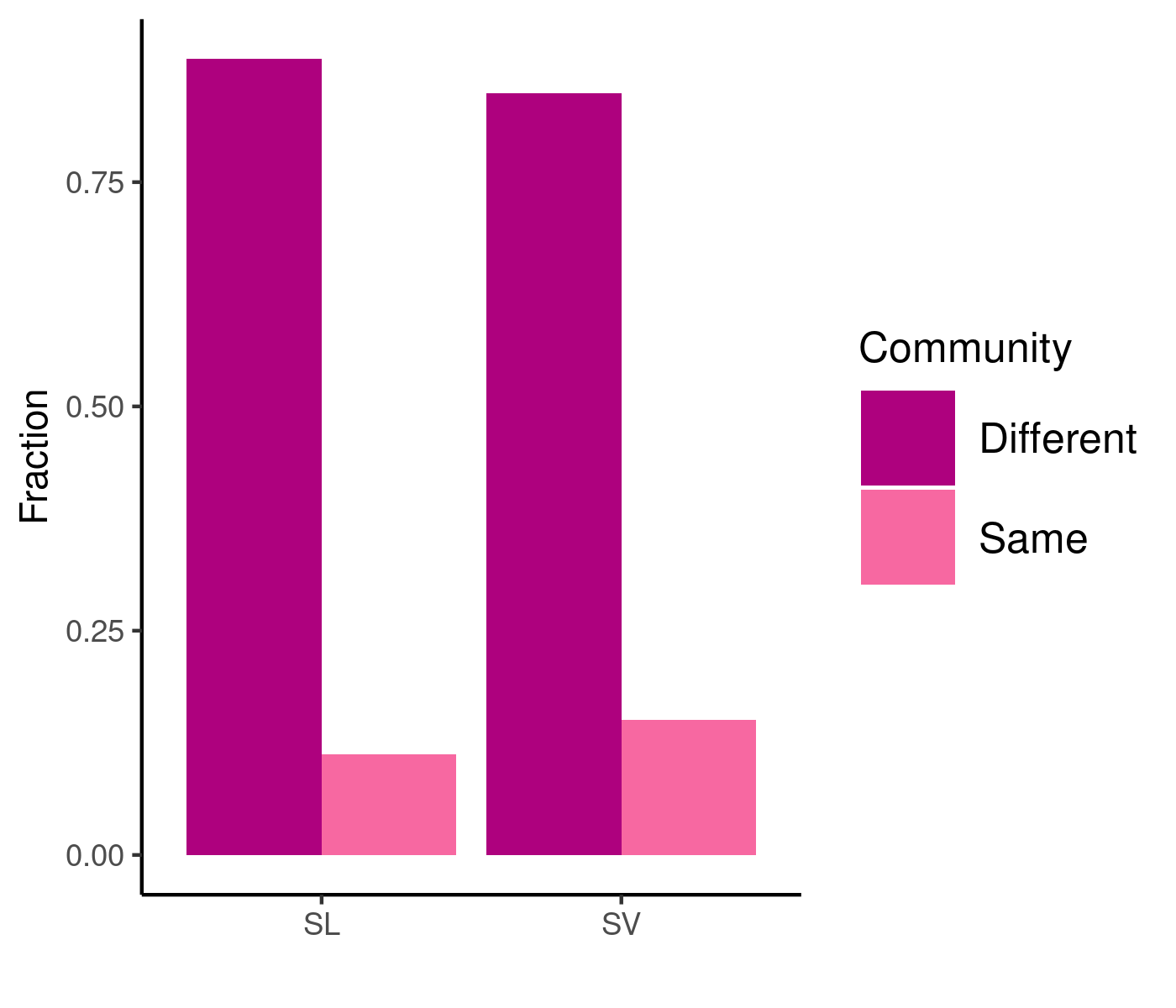

Supplement: S11 Fig — Most of the SL and SV pairs belong to different communities, and there is a difference in the proportion of SL and SV in different communities (p-value 2.2e-16, two-proportions z-test). (TIFF) [file pcbi.1012336.s011.tiff]
