# Supplementary material for: MAGICAL: A multi-class classifier to predict synthetic lethal and viable interactions using protein-protein interaction network
Source: PLoS Comput Biol. 2024 Aug 26;20(8):e1012336. doi: 10.1371/journal.pcbi.1012336 (PMC12529998; doi:10.1371/journal.pcbi.1012336)
Supplement: Attachment — Submitted filename: PCOMPBIOL-D-24-00318_Review.docx [file pcbi.1012336.s014.docx]

**Summary**

In this study, the authors trained a multi-class model to predict synthetic lethality (SL) and synthetic viability (SV) genetic interactions. The newly trained model was compared to and outperformed previously published binary classifiers. Identification of these interactions may impact oncological treatment in patients undergoing targeted therapy. While the authors highlight the importance of this research, there are major concerns that need to be addressed.

**Overall**

The manuscript seemed to be disorganized throughout and contained many grammatical errors. The introduction is two very long paragraphs that essentially repeat themselves. Additionally, a large part of the introduction describes the downfall of using machine/deep learning models, particularly the decipherability, but then proceeds to use a machine learning model for their classifier.

The Materials & Methods section, however, is the most discombobulating. The *Training and Validation dataset* section does not clearly describe how many patients/demographic information/disease types of either of the two datasets mentioned in this section. Additionally, a third dataset is described after and lacks this information. The following sections furthermore seem out of place: *Network Analysis* and *Selection of ML model* are not clear as to their purposes and may be combined for clarity. Additionally, *Balancing the imbalance dataset* is well described but should be regarded before network analysis as class balance should have been completed before model training. If class balancing was completed after training, the authors should consider retraining their model.

The statistical analysis plan was never discussed in the paper. Results were described using accuracies and feature importance, and then later described using Kolmogorov-Smirnov p-values. The results section also describes several instances of methodology that would have made the set-up of these sections far more understandable. The authors also use language such as “perform well” or “better” than other models without using statistical measures of those statements in the text.

**Figures**

- Supplemental Figures 1 and 2 are flipped.
- Figure 6 should be in the results section, not discussion.
- All figures should contain significance bars to prove the value of what is being shown.
- Figures need clear labels and legends. A figure should be able to tell you the results without needing the aid of the long figure captions.
- Figure 4 is very confusing. What do the three colors within each of the bar graphs represent?
