# Supplementary material for: MAGICAL: A multi-class classifier to predict synthetic lethal and viable interactions using protein-protein interaction network
Source: PLoS Comput Biol. 2024 Aug 26;20(8):e1012336. doi: 10.1371/journal.pcbi.1012336 (PMC12529998; doi:10.1371/journal.pcbi.1012336)
Supplement: Attachment — Submitted filename: magical-review-comments-24-06-24.docx [file pcbi.1012336.s015.docx]

**Reviewer #1:**

The work is very good, simple, direct and with an important contribution to the area. I just want to note that when showing the ROC curves, it is important to indicate the confidence intervals of the AUC values and, if possible, perform a DeLong test to compare them and indicate significant differences,and then discuss the results, taking into consideration these differences demonstrated by formal statistical methods. Because the ROC method usually uses bootstraps, confidence intervals can be obtained by randomizing the bootstrap.

Thank you for reviewing our work. We calculated the confidence interval and noted that 90% confidence interval for the AUC on the test data set is between 0.897-0.913. We performed the DeLong test to compare the AUC values of SLant and MAGICAL for both balanced and unbalanced data. In every comparison, the AUC value of MAGICAL is significantly more than SLant (p-value 0.0003, < 2.2e-12, respectively, DeLong test). The p-value is shown in Figure 5A, B legend, page number 16, line number 327-330. We have also added confidence intervals of AUC values in the manuscript. (Page number 14, line number 296)

**Reviewer #2:**

Summary

In this study, the authors trained a multi-class model to predict synthetic lethality (SL) and synthetic viability (SV) genetic interactions. The newly trained model was compared to and outperformed previously published binary classifiers. Identification of these interactions may impact oncological treatment in patients undergoing targeted therapy. While the authors highlight the importance of this research, there are major concerns that need to be addressed.

Overall

The manuscript seemed to be disorganized throughout and contained many grammatical errors. The introduction is two very long paragraphs that essentially repeat themselves. Additionally, a large part of the introduction describes the downfall of using machine/deep learning models, particularly the decipherability, but then proceeds to use a machine learning model for their classifier.

Thank you for reviewing our work. We apologize for the lengthy introduction and the grammatical errors. We have cut short the introduction part and have summarized it more precisely. We fail to agree that the manuscript describes the downfall of machine learning methods. In the introduction, we discussed the limitations of a binary classifier and how having a binary classification does not correctly classify a synthetic lethal interaction from a mixture of other genetic interactions and pairs that do not interact. We have improved the texts and have tried our best to communicate the study well.

The Materials and Methods section, however, is the most discombobulating. The Training and Validation dataset section does not clearly describe how many patients/demographic information/disease types of either of the two datasets mentioned in this section. Additionally, a third dataset is described after and lacks this information.

This study does not involve any patient data for model building; thus, patients/demographic information/disease types have not been mentioned. The data for Kaplan Meier plots shown in Figures 5D and E are retrieved from the TCGA pan-cancer study. For both NPRL2-EP300 and GCN1-LDLR pairs, datasets from **2683** samples/**2565** patients are used. The number of patients' information has been included in the methodology and result section and can be found on pages 7 and 17 and lines 169 and 352, respectively.

The following sections furthermore seem out of place: Network Analysis and Selection of ML model are not clear as to their purposes and may be combined for clarity.

Thank you for your suggestion; the network analysis and selection of ML models are two different analyses and, thus, have been kept under two different sub-headings for better understanding and clarity. But we have changed it as per the suggestion. Please refer to page numbers 8 and 10 and line numbers 185 and 212, respectively.

Additionally, Balancing the imbalance dataset is well described but should be regarded before network analysis as class balance should have been completed before model training. If class balancing was completed after training, the authors should consider retraining their model.

The balancing of the dataset was completed before the model's training and has now been added before the model discussion. Please refer to page number 7, line number 179.

The statistical analysis plan was never discussed in the paper. Results were described using accuracies and feature importance, and then later described using Kolmogorov-Smirnov p-values. The results section also describes several instances of methodology that would have made the set-up of these sections far more understandable. The authors also use language such as “perform well” or “better” than other models without using statistical measures of those statements in the text.

Thank you for the suggestion. We have included a separate section as statistical analysis in the method section and mentioned all the statistical tests performed for the study (Page number 11, line number 248).

We have also rephrased the sentences and have included statistical tests wherever needed.

Figures

Supplemental Figures 1 and 2 are flipped.

We apologize for the mistake. The figures have been properly formatted and placed.

Figure 6 should be in the results section, not discussion.

We have now moved Figure 6 and the text linked to it to the result section.

All figures should contain significance bars to prove the value of what is being shown.

We have performed statistical tests wherever possible and added the p-values associated with them in the legend section and the manuscript text.

Figures need clear labels and legends. A figure should be able to tell you the results

without needing the aid of the long figure captions.

Thank you for your suggestion. We apologize for the confusion. We have rephrased and summarized the figure captions.

Figure 4 is very confusing. What do the three colors within each of the bar graphs

Represent?

The three colors, pink, cyan, and gray, represent the interaction types, SL, SV, and NOT, respectively, as discussed in the manuscript. The barplot represents the number of pairs correctly predicted by MAGICAL. The ROC plot shows that for a lower false positive rate value, the model could achieve a high true positive rate for all three prediction classes. For example, pink, cyan, and gray lines, respectively, show the sensitivity of MAGICAL in predicting SL, SV, and NOT pairs correctly.

**Reviewer #3:**

The authorss propose a model named MAGICAL, a multi-class machine learning model, predicts SL and SV using network properties of proteins. It achieves ~80% accuracy on training data and performs well on experimental datasets. The work has potentials, however, I have the following suggestions/comments:

-AUCROC must be calculated/plotted by more than one running point(TPR,FPR). The one in the manuscript only have one point.

Different ROC plots are generated for different cutoff/threshold values. The accuracy ranges from 84.57% to 75.71% with a running point from 0.1 to 0.9 (Supplementary Figure 8), showing high true positive rate for low false positive rate for different running points. (Page number 14, line number 297-299)

-some parameters values you need to mention why selected them e.g. why 100 bootstraps? justification is needed.

The 100 bootstraps were selected based on published works. The consideration of 100 bootstraps was also to reduce the computational time. However, now we have incorporated 1000 bootstraps and interestingly observed that the model accuracy was greater than 80% in 1000 models, and the results remained similar to the result obtained by 100 bootstraps (now Figure S5). For the top five determinant features, it was observed that average betweenness and average triangle emerged as the third and fourth important attributes for 1000 bootstraps, which were fourth and third for 100 bootstraps. Page number 12, line number 257, Figure 2. The figure below corresponds to 1000 (A) and 100 bootstraps (B).

-How the model avoided overfitting. The authors may plot the training versus validation accuracy versus multiple running points (epochs) and check.

To check this, we built four random forest models with differing numbers of trees. We notice that the accuracy for all these models does not change much with the number of trees. The accuracies for 100, 200, 500, and 5000 trees are 80.20, 80.26, 80.41, and 80.47 respectively. We also note that the error rate decreased to 100 trees, followed by a saturation after 100 trees. This suggests that the model is well trained and increasing the number of trees beyond 100 does not affect the accuracy of the random forest model. Page 14, line number 304-306.

-KEGG pathway analysis and go-enrichment can be used to analyze the findings.

Thank you for the suggestion. We have performed a gene ontology-based analysis on both SL and SV pairs. It was observed that SL pairs are associated with more GO terms than SV pairs (p-value < 2.2e-16, KS test). This was followed by calculating the Jaccard index, a metric of intersection divided by union. Interestingly, it was observed that SV pairs have a higher value of the Jaccard index compared to SL pairs, suggesting that SV pairs, although involved in fewer functions, are more redundant than SL pairs (p-value 3.198e-12, KS test) (Figure 6B, C, page number 18, line number 374-376).

For example, BRCA1 and VEGFA, an SL interaction, are associated with 60 and 167 GO terms, respectively. Surprisingly, a poor overlap of 3 GO terms indicates that this pair has a lot of unique GO terms of different and diverse roles. The common GO terms, "GO:0010628", "GO:0045766", and "GO:0045944" are associated with distinct biological roles, such as positive regulation of gene expression, positive regulation of angiogenesis, and positive regulation of transcription by RNA polymerase II, respectively. BRCA1 and VEGFA, although an SL pair, seem to be involved in two biological modules. Interestingly, for an SV interaction, MED12 and MED14, an overlap of 4 out of a total of 25 GO terms is observed. MED12 and MED14 are subunits of the mediator complex that regulate RNA polymerase II and function as a transcription coactivator. The GO terms “GO:0045944” and “GO:0060261” are associated with “positive regulation of transcription by RNA polymerase II”. Thus, SV interactions tend to participate and share more biological processes than the SL pairs. In contrast, SL pairs reside in different modules, sharing fewer biological processes.

- The introduction uses suggest and suggested, I wish to unify the grammar.

Thank you for the suggestion, we have unified the grammar wherever needed.
