# Supplementary material for: MAGICAL: A multi-class classifier to predict synthetic lethal and viable interactions using protein-protein interaction network
Source: PLoS Comput Biol. 2024 Aug 26;20(8):e1012336. doi: 10.1371/journal.pcbi.1012336 (PMC12529998; doi:10.1371/journal.pcbi.1012336)
Supplement: Attachment — Submitted filename: MAGICAL-2nd-review-comments-15-07-24.docx [file pcbi.1012336.s016.docx]

Reviewer #2: While the paper has significantly improved from the initial review, there is still lacking information that is critical for understanding.

1. The train/test split is still not fully explained. I understand that SLant was trained on the 411 SL and 411 non-SL, but what was MAGICAL trained on? How many samples in the training data? How many samples in the test data?

The sample size of SL, SV and NOT used to train MAGICAL was shown in Figure 1. Thank you for the suggestion to include it in the main text. MAGICAL is trained on three datasets, CGIdb, BioGRID, and SynLethDB, with a total of 26,445 SL, 3,867 SV, and 9,986 NOT interactions after data preprocessing and mapping the pairs to the network (now included in page number 6, line number 153). As discussed, SMOTE was used to balance the data, resulting in 11,238 SL, 11,601 SV, and 4,230 NOT data, totaling 27,069 pairs. This data is divided into a 70-30 ratio: 70% (18,948) was used for training and 30% (8,121) for testing. The numbers are now included in page 8, line numbers 195-198.

2. Was SLant employed on the data used in this study? Or were the predictions taken from the cited papers?

Due to the unavailability of the SLant model, we developed a binary class model (SLant) utilizing network property values (calculated by us for BioGRID protein-protein interaction network) for the SL pairs reported in BioGRID. SLant, as mentioned in Benstead et al., has been trained on BioGRID data consisting of 411 SL and 411 non-SL interactions. We retrieved 411 SL pairs from BioGRID and 411 non-SL from our ‘NOT’ data. The determinant network properties as features (coreness, adhesion, and cohesion) mentioned by Benstead *et al.,* were utilized in training this binary class model (SLant). We compare the accuracy of this binary model (SLant) to that of the MAGICAL, which is trained on five determinant network properties (shortest path, average neighbor2, average betweenness, average triangle, and adhesion) and establish the efficiency of MAGICAL over SLant, (now included in page number 7, line number 159-169).

3. Why were Kaplan Meier curves only plotted using TCGA data and not using the training dataset?

The training dataset includes genetic interactions retrieved from CGIdb, BioGRID, and SynLethDB. No patient data, such as survival, has been used for the training. We plotted KM plots to compare the survival of the patients harboring alteration in predicted SL/SV pairs with the unaltered groups as reported in TCGA. By definition, patients harboring alteration in SL pairs should have better survival than the unaltered group. Similarly, patients harboring alteration in SV pairs would have poorer survival than the unaltered group. For example, the survival of patients with mutations in both GCN1-LDLR genes is better than that of patients with no mutation indicating SL interaction. Similarly, the survival of patients with a mutation in both NPRL2-EP300 genes is worse than those with no mutation indicating SV interaction (page number 17-18, line number 367-373). Figure 5D, E.

4. Statistical Analyses section needs to be further expanded explain why/how tests are chosen. As stands, it is lacking information to reproduce results.

As mentioned, different tests, such as the Kolmogorov-Smirnov (KS) test and the DeLong test, have been performed to provide statistical significance to the analysis carried out in the study. The KS test has been carried out to test the differences in the distribution of network property values for all three classes: SL, SV, and NOT. The DeLong test is performed to test the difference in the accuracy between the two models. We employed the *ks.test* function in R to establish the significant differences in the distribution of the genetic interactions. For the DeLong test *roc.test* function in R with the “delong” method was used. The same has been included now in the main text on page number 12, line number 262.

Figures:

1. In general, make sure titles are on plots where it is not readily apparent what the graph is showing.

Thank you for the suggestion. We have now added titles wherever needed.

2. Figure 5: it would be helpful to display p-values in KM plot similarly to AUCs in ROC plots.

The updated figure shown below has been included in the manuscript.

3. Figure 6: node colors should be consistent with the colors used in other plots

Thank you for the suggestion. The same has been changed to maintain consistency.

4. Supp. Figure 2: x-axis label is not correct. Please adjust.

We apologize for this mistake. The revised figure with the correct x-axis label has been included in the supplementary file.

Supplemental tables 1 and 2 do not appear in the supplemental document (if submitted as separate files, disregard).

We have submitted the tables as zip files.

Reviewer #3: The authors addressed the reviewers concerns. The manuscipr is in a very good shape for publication.

Thank you very much for your response. All the authors are grateful for your help in improving the quality of our work.
